# Chronic Nasal Catarrh—A Simple and Effective Treatment

**Published:** 1904-05

**Authors:** G. A. Gilbert

**Affiliations:** Danbury, Conn.


### CHRONIC NASAL CATARRH.—A SIMPLE AND EFFECTIVE TREATMENT.

By G. A. GILBERT. M.D., DANBURY, CONN.

We feel inclined to report this case here not only because of the marked and speedy results obtained from the simple plan of treatment adopted after the usual measures had been tried and proven ineffective, but because of the frequency with which the particular group of symptoms complained of by this patient confronts the general practitioner in his everyday work.

Lena D—, a young miss of eighteen, had been a sufferer from chronic rhinitis or pharyngo-nasal catarrh for more than ten years, being subject to periodical attacks of coryza and tonsillitis, especially during the spring, fall and winter months. The mucus membrane of throat and nose became habitually flabby, congested and swollen.

At the age of twelve, the characteristic thick, indistinct speech and stertorous breathing of the catarrhal subject became manifest, while at the same time, the plugging of the pharyngeal opening of the eustachian tube by the thickened mucus secretions gave rise to slight deafness.

The treatment throughout had consisted of insufflations of the usual antiseptic powders, ante- and post-nasal douches with the modern germicidal solutions, while various astringent or disinfectant gargles and sprays were used for the tonsillitis, but these gave only temporary relief. It was apparent that only the membranous surface was thus freed of its obnoxious discharges and not the deeper sub-mucus tissues and gland sacs which harbored (unwillingly) the germs that gave birth to these discharges, and it became self-evident that some more active method of treatment must be adopted.

In dental surgery, it is well known that an antiseptic solution having an alkaline base is the most effective for cleansing the mouth of putrefactive material arising from fermented food (starch particles in the substances adhering to the teeth), as well as that caused by the bacteria of dental caries, leptothris buccalis, etc. This fact is explained on the chemical ground that the alkaline base combines readily with these various weak acids with which it comes in contact, thus breaking up the solution and liberating the oxygen or oxidizing agent upon which its disinfectant properties depend. In a word, such an alkaline agent dissolves the mucus secretions, and weak acids which form in the mouth.

Were the foregoing all that is required of an antiseptic, nothing further would need to be said, but it is essential that the bacteria hidden more deeply within the walls of the gland sacs should also be removed. Recognizing the force of the suggestion recently made by scientific investigation, i. e., that a true alkaline germicide dissolves the bacterial envelope instead of coagulating it as do the acids and that if the specific gravity is favorable to low exosmotic action it will be absorbed into the surrounding tissues and gland sacs, where the germs are hidden, it at once occurred to us that an alkaline agent of this character was just what was needed.

Feeling convinced that an alkaline antiseptic was strongly indicated in this case, the best of its kind, Glyco-Thymoline, being selected, was applied thoroughly once every day by myself and three or four times daily by the patient. A 25 per cent. watery solution (warm) of Glyco-Thymoline was made by me and applied in a fine spray to the post-nasal chamber by means of a hand atomizer. The nozzle was turned up at the end so that when introduced well back into the pharynx the spray was thrown upward direct into the post nares.

The patient herself soon learned to operate the post-nasal douche satisfactorily and was instructed to spray the parts in this manner twice daily, besides applying the solution (in the same strength), with the K. & O. douche. At the same time an ounce of a 50 per cent. solution of Glyco-Thymolene was gargled and used as a mouth wash three times daily for the purpose of hardening the flabby, congested tonsils.

The outcome of this simple plan of treatment soon made plain the fact that a germicidal agent was being employed in this case, which possessed the alkaline and solvent properties already mentioned as being essential to success. The patient's general system had first been thoroughly purged of retained waste by way of kidneys and bowels, after which the local treatment was adopted as above described. This latter procedure was not only effective, but the antiseptic proved very agreeable to the patient who, for the first time in several years, experienced the sensation of possessing a clean, sweet mouth.

The hypertrophied membrane itself grew almost normal in appearance, distinctness of speech and hearing was gradually restored, the breathing became natural, and at the end of three months we had accomplished a speedy and perfect cure.

